# The Apoptosis Effect on Liver Cancer Cells of Gold Nanoparticles Modified with Lithocholic Acid

**DOI:** 10.1186/s11671-018-2653-8

**Published:** 2018-09-29

**Authors:** Mei-Xia Zhao, Zhong-Chao Cai, Bing-Jie Zhu, Zhi-Qiang Zhang

**Affiliations:** Key Laboratory of Natural Medicine and Immune Engineering, Henan University, Kaifeng, 475004 China

**Keywords:** Gold nanoparticles, Lithocholic acid, Liver cancer cells, Apoptosis

## Abstract

Functionalized gold nanoparticles (AuNPs) have widely applied in many fields, due to their good biocompatibility, a long drug half-life, and their bioactivity is related to their size and the modified ligands on their surface. Here, we synthesized the AuNPs capped with ligands that possess polyethylene glycol (PEG) and lithocholic acid (LCA) linked by carboxyl groups (AuNP@MPA-PEG-LCA). Our cytotoxicity results indicated that AuNP@MPA-PEG-LCA have better cell selectivity; in other words, it could inhibit the growth of multiple liver cancer cells more effectively than other cancer cells and normal cells. Apoptosis plays a role in AuNP@MPA-PEG-LCA inhibition cell proliferation, which was convincingly proved by some apoptotic index experiments, such as nuclear staining, annexin V-FITC, mitochondrial membrane potential (MMP) analysis, and AO/EB staining experiments. The most potent AuNP@MPA-PEG-LCA were confirmed to efficiently induce apoptosis through a reactive oxygen species (ROS) mediating mitochondrial dysfunction. And AuNP@MPA-PEG-LCA could be more effective in promoting programmed cell death of liver cancer cells.

## Background

Gold nanoparticles (AuNPs) as nano-materials have widely applied in many fields because of its unique optical properties, good chemical stability, and biocompatibility [1–5]. So, it has broad application prospects in nano-electronics, nano-photonics, catalysis, sensors, biomarkers, and many other areas [6–8]. Because AuNPs have large surface area and spherical shape, they can be used as carrier for antineoplastic drugs [9–12]. Moreover, many AuNP complex have been mainly used for the new type of antitumor drugs in order to treat cancer [13, 14]. As an antineoplastic drug carrier, AuNP complex can control cell function, regulate gene expression, and detect analytes in the cell [15, 16]. Therefore, the improvement of functionalized AuNPs becomes one of the important trends in the research of cancer treatment [17–19].

Lithocholic acid (LCA) widely exists in higher vertebrate secondary bile acid in the bile. Bile acid species diversity has been reported in the application of different kinds of bile acid and its derivatives in medicine and some other fields [20–22], such as it can be used in the treatment of bile acid deficiency, gallstones, and liver disease [23–25]. And some bile acids and its derivatives can as drug carriers target treatment of liver disease, absorption promoter, and lower agent of cholesterol. [26–28]. Previous reports demonstrated that LCA has a very strong antitumor effect in liver cancer cells, and the cell death mechanism is apoptosis [29, 30]. Apoptosis is a biological cell active death process, and it is an important mechanism that the multicellular organism body regulates the body development, controls cell aging, and maintains internal environment stable [31, 32]. Especially, inhibition of proliferation, differentiation, reduction of the malignant degree, and promotion of the tumor cell apoptosis are the purposes of tumor treatment [33–37].

In this study, we synthesized the AuNPs with biological targeting properties through combining gold NPs with LCA derivatives. We studied their cytotoxicity by using MTT method with HepG2, SMMC-7721, QSG-7701, and MCF-7 cells for 48 h. Our cytotoxicity results revealed that AuNP@MPA-PEG-LCA could inhibit the growth of multiple liver cancer cells more effectively than other cancer cells and normal cells. Apoptosis plays a role in inhibition cell proliferation, which was confirmed through Hoechst 33342 staining, annexin V-FITC staining, mitochondrial membrane potential (MMP) analysis, and AO/EB staining experiments. And the ROS level increased in liver cancer cells, suggesting that AuNP@MPA-PEG-LCA may induce apoptosis via ROS generation-mediated mitochondrial dysfunction.

## Methods

### Materials

Unless specified, chemicals were purchased from Sigma-Aldrich (St. Louis, MO) and used without further purification. RPMI-1640 media and fetal bovine serum (FBS) were from Invitrogen Corporation. HepG2 (human hepatocellular liver carcinoma cells), SMMC-7721(human hepatocellular liver carcinoma cells), QSG-7701 (human normal hepatocyte cells), and MCF-7 (human breast cancer cells) were purchased from the Shanghai Institute for Biological Science (Shanghai, China).

### Synthesis of AuNP@MPA

Citrate-capped gold nanoparticles (AuNP@MPA) with an average size of 4.0 nm were prepared according to the method pioneered by J. Turkevich et al. [38]. Briefly, 773 μl of 38.8 mM sodium citrate solution and 2 mL of 15 mM of HAuCl_4_ solution were dissolved to 30 mL of Milli-Q H_2_O, and the solution was stirred at 25 °C. Then, 3 mL of 0.1 M of NaBH_4_ (freshly prepared) was added. After reacting for 2 h 25 °C, the solution changed from colorless to light orange. Then, 3 mL of 0.01 M of 3-mercaptopropionic acid (MPA) in anhydrous ethanol was added at pH 11, and kept reacting for 2 h at 25 °C. The reaction mixture was centrifuged get compound AuNP@MPA.

### Synthesis of AuNP@MPA-PEG

10.5 mg (0.0875 mmol) 1-Hydroxypyrrolidine-2,5-dione (NHS) and 7 mg (0.035 mmol) 1-(3-dimethylaminopropyl)-3-ethylcarbodiimide hydrochloride (EDC) were added to 50 mM AuNP@MPA solution in 4-Morpholineethanesulfonic acid (MES), and the solution were stirred for 30 min at 25 °C. Then, 0.045 mmol NH_2_-PEG1000-NH_2_ was added and the mixture was stirred for 24 h at 25 °C. When the reaction is complete, the mixture was centrifuged to get compound AuNP@MPA-PEG.

### Synthesis of AuNP@MPA-PEG-LCA

The compound AuNP@MPA-PEG in ultrapure water was added to 200 μL anhydrous dimethylformamide (DMF) solution including 17 mg (0.045 mmol) LCA, and the reaction solution was stirred for 24 h at 25 °C. When the reaction is complete, the reaction mixture was centrifuged to get compound AuNP@MPA-PEG-LCA.

### Transmission Electron Microscopy (TEM)

The morphology and size of AuNPs were detected on a JEOL JEM-200CX TEM, operating at up to 200 kV. The AuNP solution was dropped on a copper grid (300 mesh).

### Antitumor Ability Assays of AuNPs

We used four types of cells (HepG2, SMMC-7721, QSG-7701, and MCF-7) to investigate the antitumor ability of the AuNPs by a modified 3-(4,5-dimethyl-2-thiazolyl)-2,5-diphenyltetrazolium bromide (MTT) method. The 0.2, 0.4, 0.6, 0.8, and 1.0 mg/mL of AuNPs and original gold nanoparticles (AuNP@MPA) were used in the assay. The OD570 of each well was measured on a Tecan Infinite M200 multimode plate reader.

### Determination of the Morphology by Hoechst Staining

After 24 and 48 h with AuNP@MPA-PEG-LCA (0.5 mg/mL), HepG2 cells were stained with 10 μg/mL of Hoechst 33342 for 30 min in cell incubator. The morphology of cell nuclei was detected by Leica-SP8 confocal microscopy.

### Cell Prophase Apoptosis: Annexin V-FITC Staining

After 6 h with AuNP@MPA-PEG-LCA (0.5 mg/mL), the HepG2 cells were stained with annexin V-FITC for 10 min in cell incubator, then observed with a Leica-SP8 confocal microscope.

For flow cytometer detection of AuNPs, after 6 h of treatment with AuNP@MPA-PEG-LCA (0.5 mg/mL), HepG2 cells were stained with annexin V-FITC for 10 min in cell incubator and were detected with a FACSCalibur flow cytometer (Becton Dickinson & Co., Franklin Lakes, NJ).

### Analysis of Mitochondrial Membrane Potential (MMP)

HepG2 cells treated with AuNP@MPA-PEG-LCA (0.5 mg/mL) for 6 h at 37 °C were incubated with 10 μg/mL JC-1 (5,5′,6,6′-tetrachloro-1,1′,3,3′-tetraethylbenzimidazolylcarbo-cyanine iodide; molecular probes) for 10 min at 37 °C. The cells were subsequently detected with a FACSCalibur flow cytometer.

For fluorescence microscopy observation, the HepG2 cells were treated with AuNP@MPA-PEG-LCA for 10 min with 10 μg/mL JC-1 and observed by using Leica-SP8 confocal microscopy.

### Measurement of Reactive Oxygen Species (ROS)

The accumulation of intracellular ROS was assayed using 2′,7′-dichlorofluorescein diacetate (H_2_DCF-DA). The HepG2 cells treatment with AuNP@MPA-PEG-LCA samples (0.5 mg/mL) for 6 h were incubated with 10 μM of H_2_DCF-DA for 30 min at 37 °C. And the fluorescence intensity of cells was viewed by using a FACSCalibur flow cytometer and a confocal microscope.

## Results and Discussion

### Preparation and Characterization of AuNPs

**Scheme 1:**
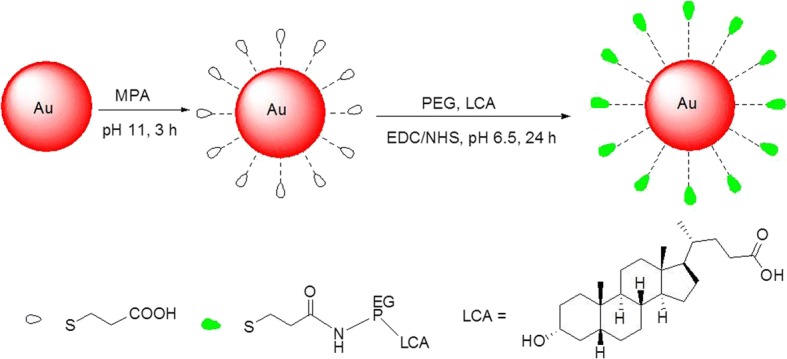
Schematic representation of synthesis of the AuNP@MPA-PEG-LCA

**Fig. 1:**
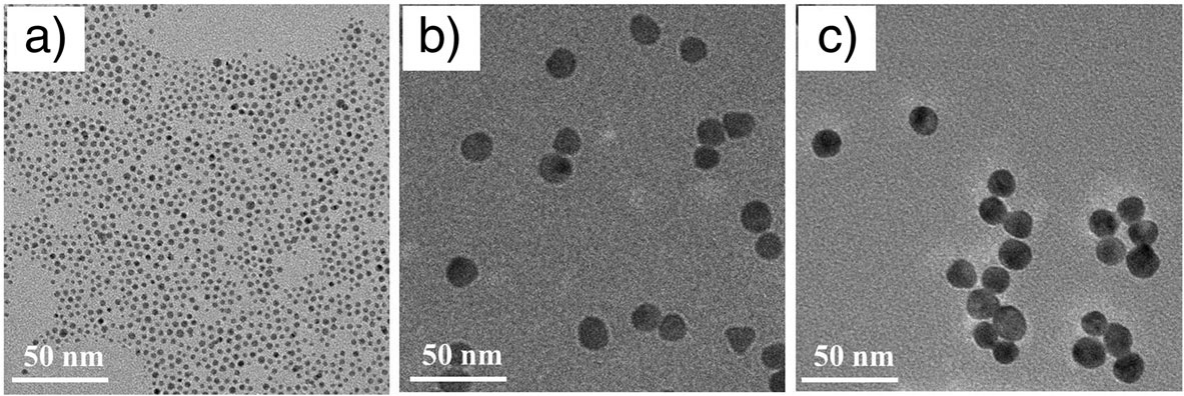
TEM images of **a** AuNP@MPA, **b** AuNP@MPA-PEG, and **c** AuNP@MPA-PEG-LCA

Firstly, water-soluble AuNP@MPA was prepared (Scheme 1). Figure 1a depicts the TEM results of the AuNP@MPA. It is indicated that the shape and size of AuNP@MPA appeared the very similar spherical shape with compact size of 4.0 ± 0.5 nm. The AuNP@MPA-PEG-LCA was then prepared (Scheme 1). And the morphology of particles was also analyzed by TEM (Fig. 1c). TEM images depicted that the morphology of AuNP@MPA-PEG-LCA is similar to that of the AuNP@MPA. And the diameters of AuNP@MPA-PEG-LCA were ~ 16 nm through statistical analysis.

### The Results of Cytotoxicity

**Fig. 2:**
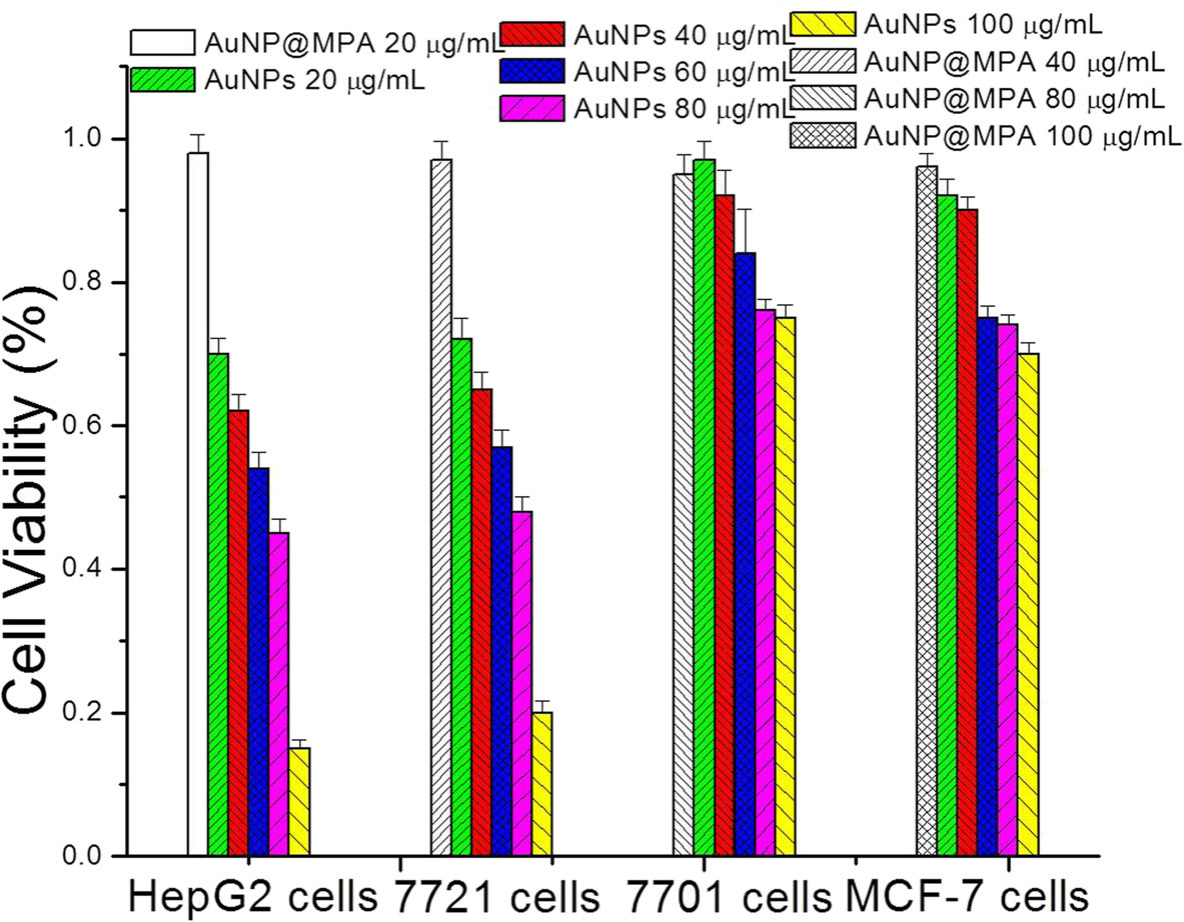
Cell viability of AuNP@MPA and AuNP@MPA-PEG-LCA (AuNPs) incubated with HepG2, SMMC-7721, QSG-7701, and MCF-7 cells for 48 h

In order to investigate the cytotoxicity of the AuNPs, HepG2, SMMC-7721, QSG-7701, and MCF-7 cells were selected and treated with AuNPs and AuNP@MPA for 48 h. And the MTT assay was used to detect the cell toxicity of samples. The cell viabilities of the AuNPs and AuNP@MPA on different cells are shown in Fig. 2. The results suggested that the cytotoxicity of AuNP@MPA is very low in all cells. However, the AuNP@MPA-PEG-LCA could inhibit the growth of multiple liver cancer cells more effectively with increasing nanoparticle concentration but have less damage to normal cells and other cancer cells. Namely, the antiproliferative activity of the AuNP@MPA-PEG-LCA to HepG2 and SMMC-7721 cells was very high relative to QSG-7701 and MCF-7 cells.

### Induction of Apoptosis

**Fig. 3:**
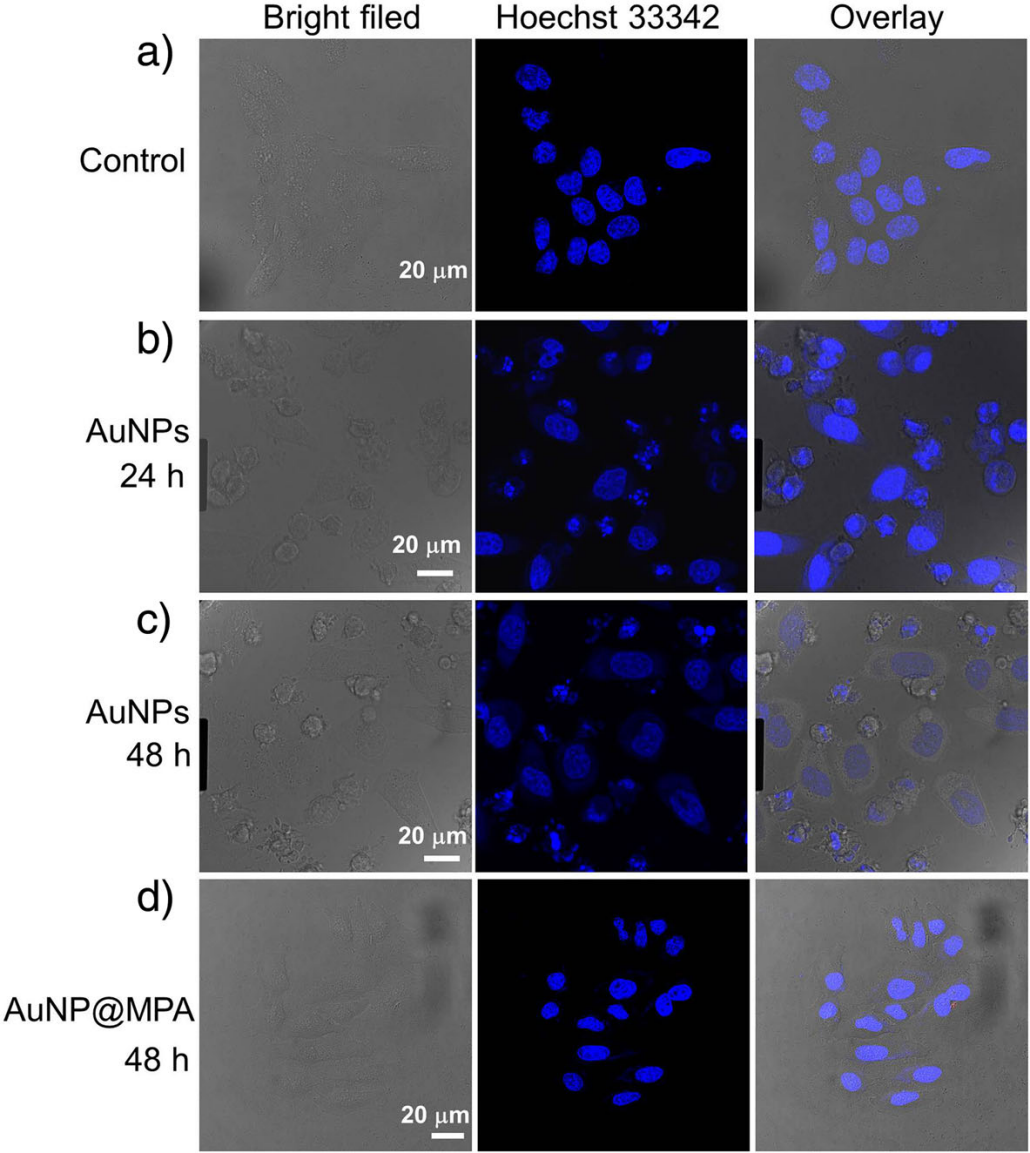
Morphologic characteristics of cell nucleus of HepG2 cells stained with Hoechst 33342. HepG2 cells were incubated with AuNP@MPA-PEG-LCA (0.5 mg/ml) for **a** 0 h, **b** 24 h, and **c** 48 h, and **d** AuNP@MPA (0.5 mg/ml) incubated for 48 h. Apoptotic cells displayed condensed and fragmented nuclei, and shrinkage of cell volume; scale bar 20 μm

Apoptotic nuclei is a common index of apoptosis. After treatment with AuNP@MPA-PEG-LCA for the indicated times, the cells were incubated with Hoechst 33342. And then the morphologic characteristics of cell nucleus were viewed using confocal microscopy. As shown in Fig. 3, the control cells and the cells incubated with AuNP@MPA exhibit intact and homogeneous cell nucleus staining; however, the number of apoptotic cells in HepG2 cells treated with AuNP@MPA-PEG-LCA increase gradually with increasing of incubating time and the cell nucleus display typical apoptosis characteristics, such as fragmented nuclei, condensed chromatin, and reduction of cellular volume.

**Fig. 4:**
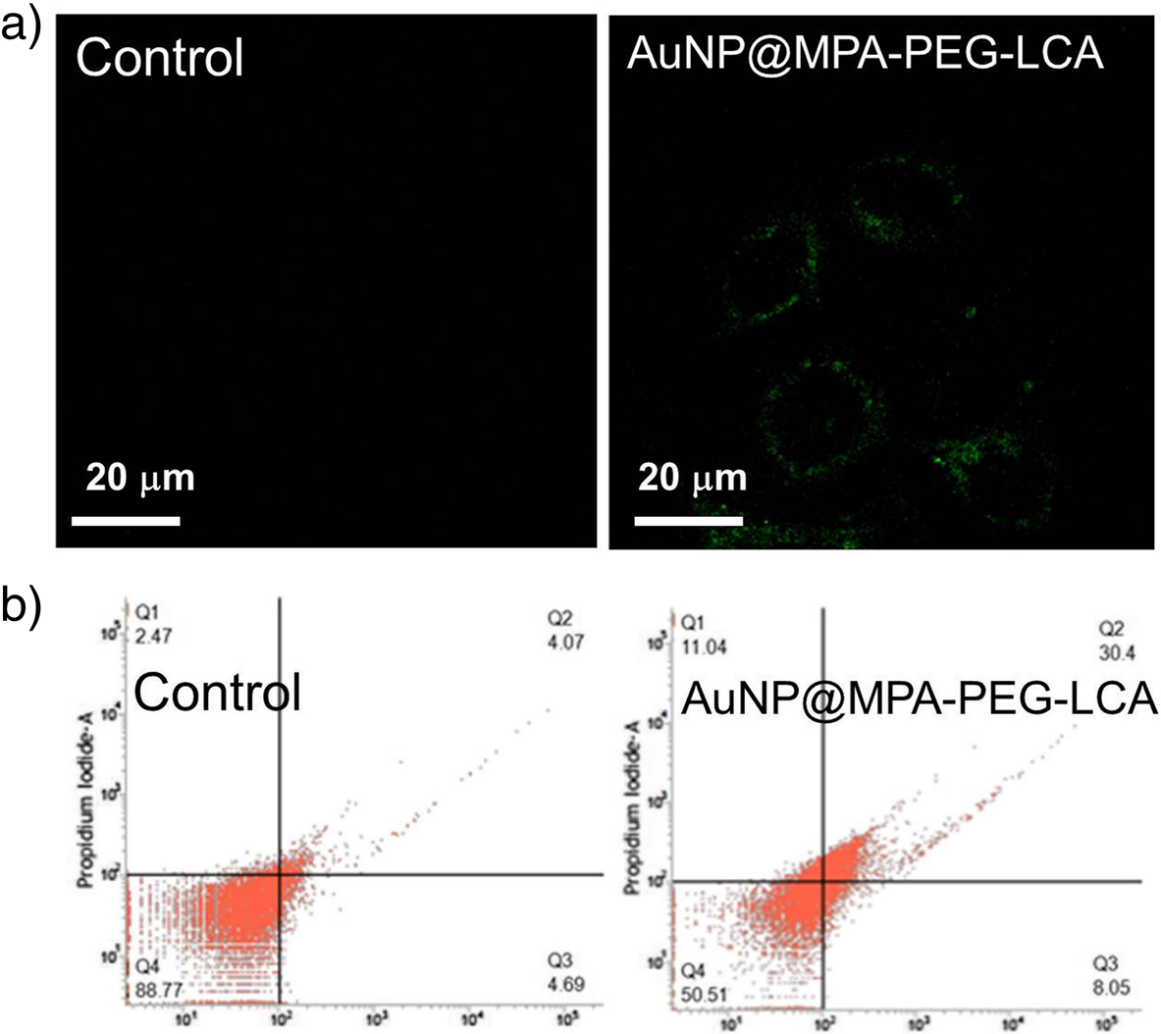
The apoptosis results of **a** confocal images and **b** flow cytometry data of HepG2 cells treated with AuNP@MPA-PEG-LCA(0.5 mg/ml). Cells were stained with annexin V-FITC (excitation at 488 nm and emission at 500–560 nm); scale bar 20 μm

As we all know, annexin V staining can distinguish the early stages of apoptosis from necrotic cells. In the early stage of apoptosis, annexin V could bind to the membrane phospholipid phosphatidylserine (PS), which is externalized from the inner to the outer surface of the plasma membrane [39]. Therefore, we investigated the potential of induce apoptosis of AuNP@MPA-PEG-LCA through annexin V-FITC staining assays. As shown in Fig. 4a, there is no obvious green fluorescence in the cell membrane in the control, but there is obvious green fluorescence in the cell membrane of HepG2 cells treated with AuNP@MPA-PEG-LCA. This phenomenon is a strong indicator of early stage apoptosis. As we all know, the images of confocal microscopy only proved the occurrence of apoptosis; however, the flow cytometry could rapidly and sensitively measure the occurrence of apoptosis and exactly determine the apoptosis rate. Therefore, we further investigated the stages of apoptosis by using flow cytometry. Figure 4b shows the results of the apoptosis rate by flow cytometry. The percentage of apoptosis was about 38.45% of HepG2 cells treated with AuNP@MPA-PEG-LCA, but the percentage of apoptosis only was about 8.16% in control cells. The significant percentage of apoptosis suggests that the cell incubated with AuNP@MPA-PEG-LCA sustained apoptosis.

### The Decrease of MMP

**Fig. 5:**
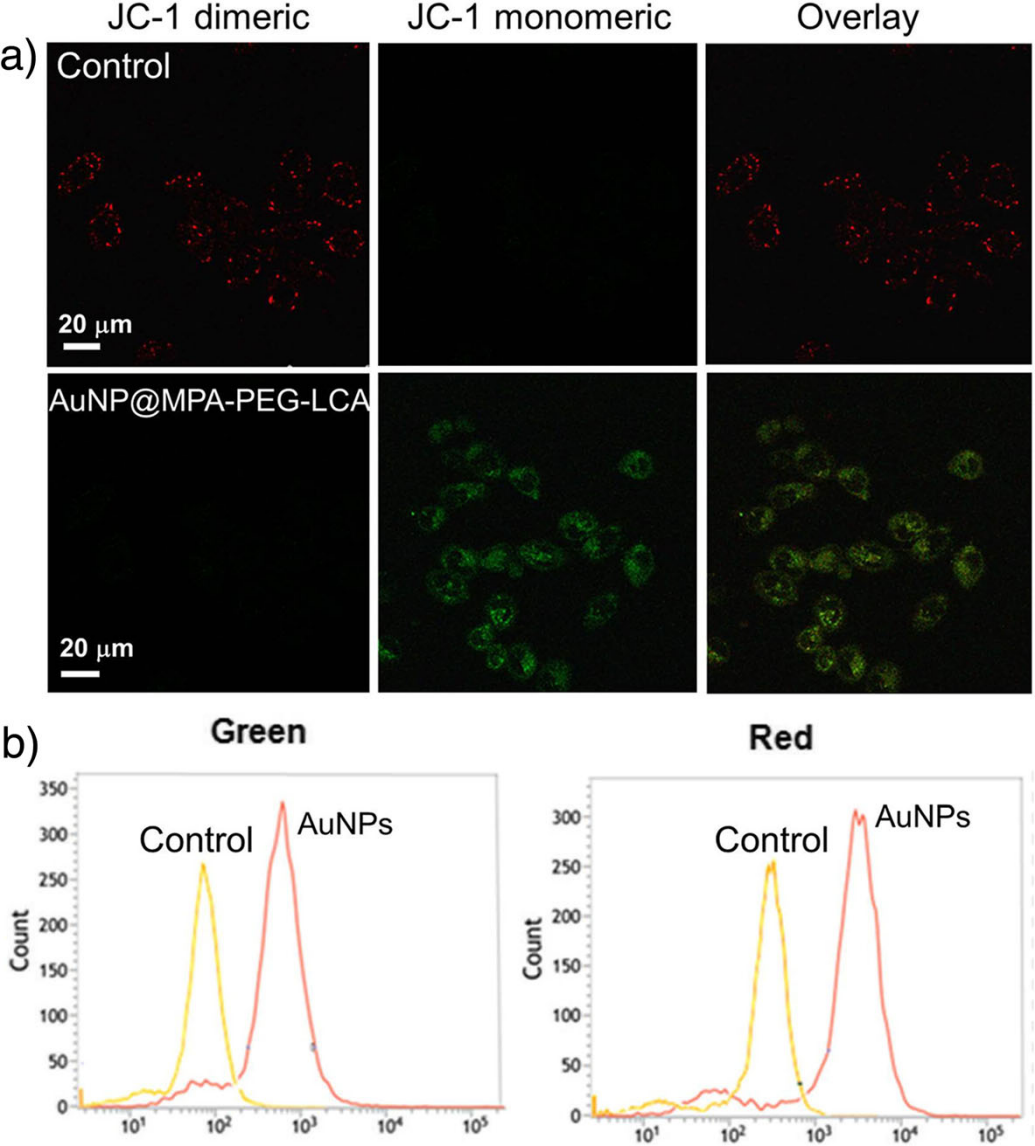
**a** Fluorescence images of JC-1 labeled cells viewed by confocal microscopy and **b** effects of AuNP@MPA-PEG-LCA on MMP analyzed by flow cytometry

Mitochondria plays an important role in apoptosis because it can release proapoptotic factors, such as cytochrome C and apoptosis-inducing factor [40–42]. So, we explored the change of MMP by using confocal microscopy and flow cytometry. Figure 5a shows the fluorescence images of JC-1 labeled HepG2 cells treated with AuNP@MPA-PEG-LCA by confocal microscopy. We can observe that there are obvious red JC-1 fluorescence and healthy mitochondria in control HepG2 cells, indicating the present of JC-1 aggregation. However, there are more green fluorescence in the HepG2 cells treated with AuNP@MPA-PEG-LCA, indicating that collapses of membrane occurred. The mitochondrial disruption in apoptotic cells indicate that the JC-1 does not accumulate inside the mitochondria but distribute throughout the cell. And as the monomeric form, the scattered JC-1 that exists fluoresces green. To further quantify the change of MMP, we evaluated the HepG2 cells stained with JC-1 by flow cytometry. Representative JC-1 red/green ratio signals recorded in HepG2 cells treated with AuNP@MPA-PEG-LCA and control cells by flow cytometry are shown in Fig. 5b. We can observe that the ratio of red/green showed a significant decrease in cells treated with AuNP@MPA-PEG-LCA from quantitative analysis of JC-1-stained cells while that had a significant increase in control cells, which suggests that AuNP@MPA-PEG-LCA can induce apoptosis in HepG2 cells.

### Effects of AuNP@MPA-PEG-LCA on ROS

**Fig. 6:**
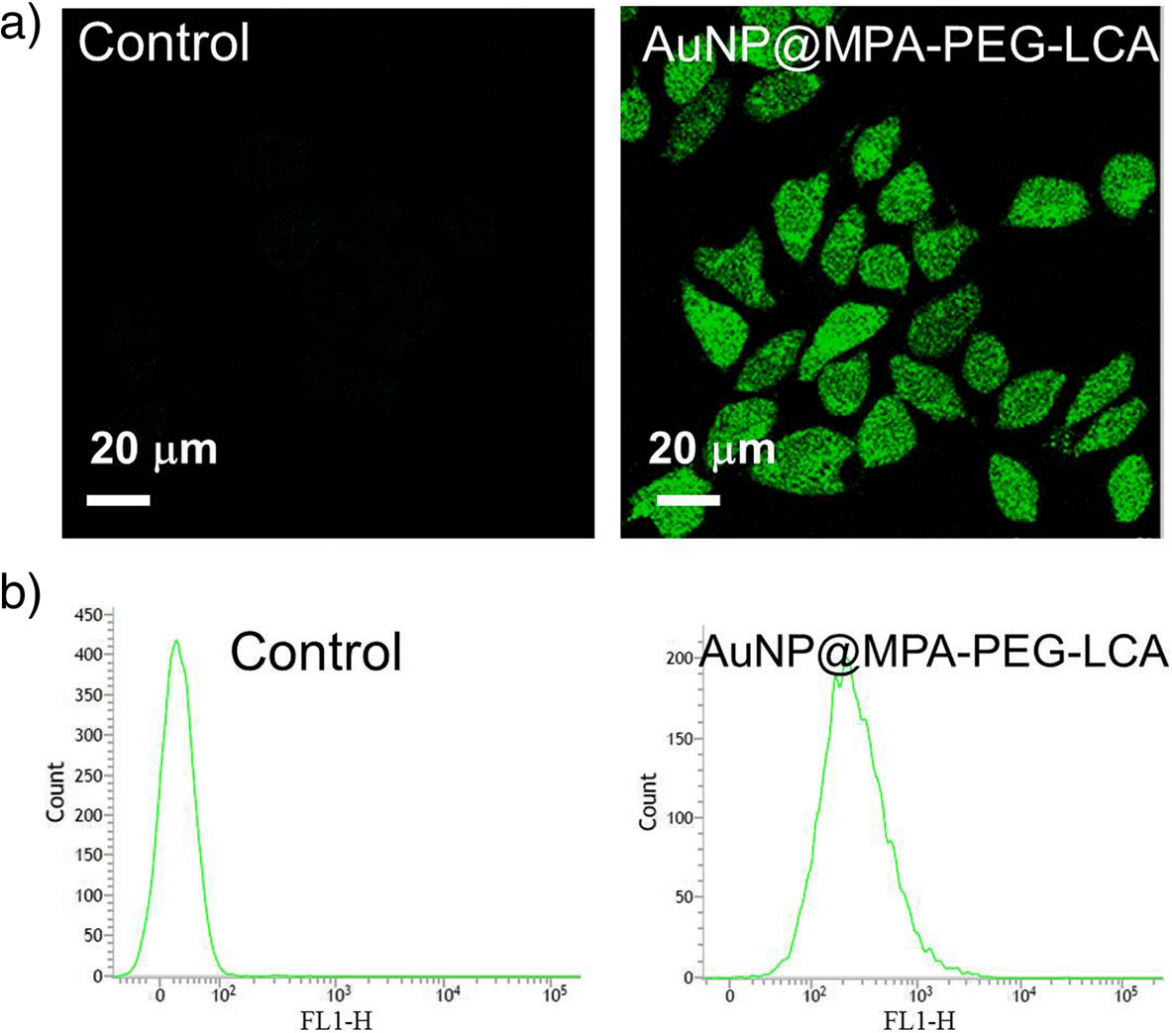
Analysis of ROS production after HepG2 cells was treated with AuNP@MPA-PEG-LCA for 6 h. The content of ROS in HepG2 cells was investigated by **a** confocal microscopy (excitation at 488 nm and emission at 530 nm) and **b** flow cytometry (excitation at 488 nm and emission at 525 nm)

As we all know, apoptosis can be triggered with increased intracellular ROS levels, which are also a strong evidence involved in the induction of apoptosis [43]. To further explore if the mitochondrial dysfunction was related to the generation of ROS, we determined the ROS level in HepG2 cells stained with H_2_DCF-DA by using confocal microscopy and the flow cytometric. As shown in Fig. 6a, the intensity of green fluorescence of H_2_DCF-DA shows a significant increase in HepG2 cells treated with AuNP@MPA-PEG-LCA compared with the control cells. That is to say, the content of ROS in HepG2 cells treated with AuNP@MPA-PEG-LCA was significantly increased. Then, the quantitative analysis of ROS content in cells was investigated by flow cytometry. As shown in Fig. 6b, the higher fluorescence intensity was detected in cells incubated with AuNP@MPA-PEG-LCA compared with the control cells, which indicates that the ROS content is higher in the cells treated with AuNP@MPA-PEG-LCA. The data suggested that the mitochondrial dysfunction was perhaps related to the generation of ROS. These results preliminarily indicate that the generation of ROS has an important role in AuNP@MPA-PEG-LCA inducing apoptosis.

## Conclusions

In summary, we synthesized the AuNP@MPA-PEG-LCA with an average diameter of 16.0 nm that can inhibit the growth of multiple liver cancer cells. The AuNP@MPA-PEG-LCA effectively inhibited the proliferation of cells due to apoptosis, which was proved by nuclear staining, JC-1 staining, MMP analysis, and annexin V-FITC staining experiments. In the flow cytometry study, AuNP@MPA-PEG-LCA arrest in liver cancer cells further proves their apoptosis behavior. Therefore, the AuNPs can efficiently induce apoptosis via a ROS-mediated mitochondrial dysfunction and they are more effective in promoting programmed cell death in liver cancer cells in a preliminary mechanistic study.

## Abbreviations

AuNPs: Gold nanoparticles
DMF: Dimethylformamide
EDC: 1-(3-Dimethylaminopropyl)-3-ethylcarbodiimide hydrochloride
FBS: Fetal bovine serum
H_2_DCF-DA: 2′,7′-Dichlorofluorescein diacetate
HepG2: Human hepatocellular liver carcinoma cells
JC-1: 5,5′,6,6′-Tetrachloro-1,1′,3,3′-tetraethylbenzimidazolylcarbo-cyanine iodide
LCA: Lithocholic acid
MCF-7: Human breast cancer cells
MES: 4-Morpholineethanesulfonic acid
MMP: Mitochondrial membrane potential
MPA: 3-Mercaptopropionic acid
MTT: 3-(4,5-Dimethyl-2-thiazolyl)-2,5-diphenyltetrazolium bromide
NHS: 1-Hydroxypyrrolidine-2,5-dione
PEG: Polyethylene glycol
PS: Phosphatidylserine
QSG-7701: Human normal hepatocyte cells
ROS: Reactive oxygen species
SMMC-7721: Human hepatocellular liver carcinoma cells
TEM: Transmission electron microscopy
